# Supplementary material for: Content-rich biological network constructed by mining PubMed abstracts
Source: BMC Bioinformatics. 2004 Oct 8;5:147. doi: 10.1186/1471-2105-5-147 (PMC528731; doi:10.1186/1471-2105-5-147)
Supplement: Additional File 2 — The original results of the above study (non-essential files are deleted to keep the file size under the limit set by BMC bioinformatics). [file 1471-2105-5-147-S2.bz2 › chilibotAdditionalFile2/dip05/704ID10734142E18041/html/MUTL_MUTS.html]

 


 **MUTL** and **MUTS** 
  
Found 186 abstracts in PubMed, retrieved 05.  
 

 What does Google say? 
 PDF only 
| .edu only 

---

**Interactive relationship** (e.g. stimulation, inhibition, etc)

**Neutral relationship**- Both pathways require the formation of a  **MutS**  homolog complex which interacts with a second heterodimer, composed of two  **MutL**  homologs.  Ref: 12836196 Med Sci Paris , 2003

**Non-interactive relationship** (e.g. studied together, co-existance, homology, etc.)

- Protein components responsible for these reactions are highly conserved through evolution, and homologs of bacterial  **MutS**  and  **MutL** , which are key players in the initiation steps of both the strand specific mismatch correction and MMR dependent apoptotic signaling, have been identified in human cells.  Ref: 12700127 Front Biosci, 2003
- As compared to native  **MutS** , the ability of MutS800 to promote  **MutL**  dependent activation of MutH is substantially reduced.  Ref: 12829697 J Biol Chem< MedlineTA>J Biol Chem, 2003
- In eukaryotes, homologs of the Escherichia coli  **MutS**  and  **MutL**  proteins are crucial for both meiotic recombination and post replicative DNA mismatch repair.  Ref: 12836196 Med Sci Paris , 2003
- Exonuclease 1 Exo1 is a 5 3 exonuclease that interacts with  **MutS**  and  **MutL**  homologs and has been implicated in the excision step of DNA mismatch repair.  Ref: 12629043 Genes Dev, 2003
- Strand specificity and repair efficiency are largely independent of the GATC methylation state of the DNA and presence of the products of mismatch repair genes mutH,  **mutL** , and  **mutS** .  Ref: 12692132 J Biol Chem< MedlineTA>J Biol Chem, 2003
